# Rash from the past: A case of recurrent reactive infectious mucocutaneous eruption triggered by common coronavirus

**DOI:** 10.1016/j.jdcr.2024.02.013

**Published:** 2024-03-01

**Authors:** Jeffrey A. Lowell, Jervon Wright, Samuel Eisenberg, Jacob Teperman, Manile Dastagir

**Affiliations:** Department of Internal Medicine, Hofstra Zucker School of Medicine and North Shore University Hospital-Long Island Jewish Medical Center at Northwell Health, Manhasset, New York

**Keywords:** adult, coronavirus, COVID-19, MIRM, *Mycoplasma pneumoniae*, reactive infectious mucocutaneous eruption, recurrent, RIME

## Introduction

Human coronaviruses cause respiratory illness, though polymorphic mucocutaneous findings can develop.^1^ Reactive infectious mucocutaneous eruption (RIME) is characterized by mucocutaneous eruptions involving 1 or more mucous membranes with limited additional cutaneous involvement. Diagnosis requires 7 to 10 days of prodromal symptoms with clinical, radiographic, or laboratory evidence of a respiratory infection, and a noncontributory medication history.^2^ While *Mycoplasma pneumoniae*–induced rash and mucositis (MIRM) was recognized in 2015, RIME was recognized after realization that other infections including *Chlamydia pneumoniae*, influenza, enterovirus, rhinovirus, metapneumovirus, and parainfluenza cause MIRM-like findings. Descriptions of RIME in the literature have increased in frequency with the emergence of severe acute respiratory syndrome coronavirus-2 (SARS-CoV-2), a novel pathogenic trigger of RIME. However, there is limited description of recurrent RIME episodes caused by nonpandemic lineage coronaviruses. Further, recurrent episodes of RIME are not well characterized. Here, we present a case in a young-adult male of recurrent RIME secondary to common coronavirus responding to intravenous immunoglobulin (IVIG) and corticosteroids.

## Case report

A 27-year-old male with prior SARS-CoV-2 infection developed pharyngitis, oropharyngeal ulcers, nonpruritic and painful rash of the extremities, injected conjunctiva, and fevers. His prodromal upper respiratory infection symptoms persisted 4 to 5 days before oral mucosal ulcers appeared. He had 2 prior episodes of self-resolving oral ulcers, once after infection with SARS-CoV-2 3 years prior, and after an unidentified viral-like illness treated with amoxicillin 3 months before this hospitalization. His only other medication exposure was valacyclovir, after developing skin lesions.

**Fig 1:**
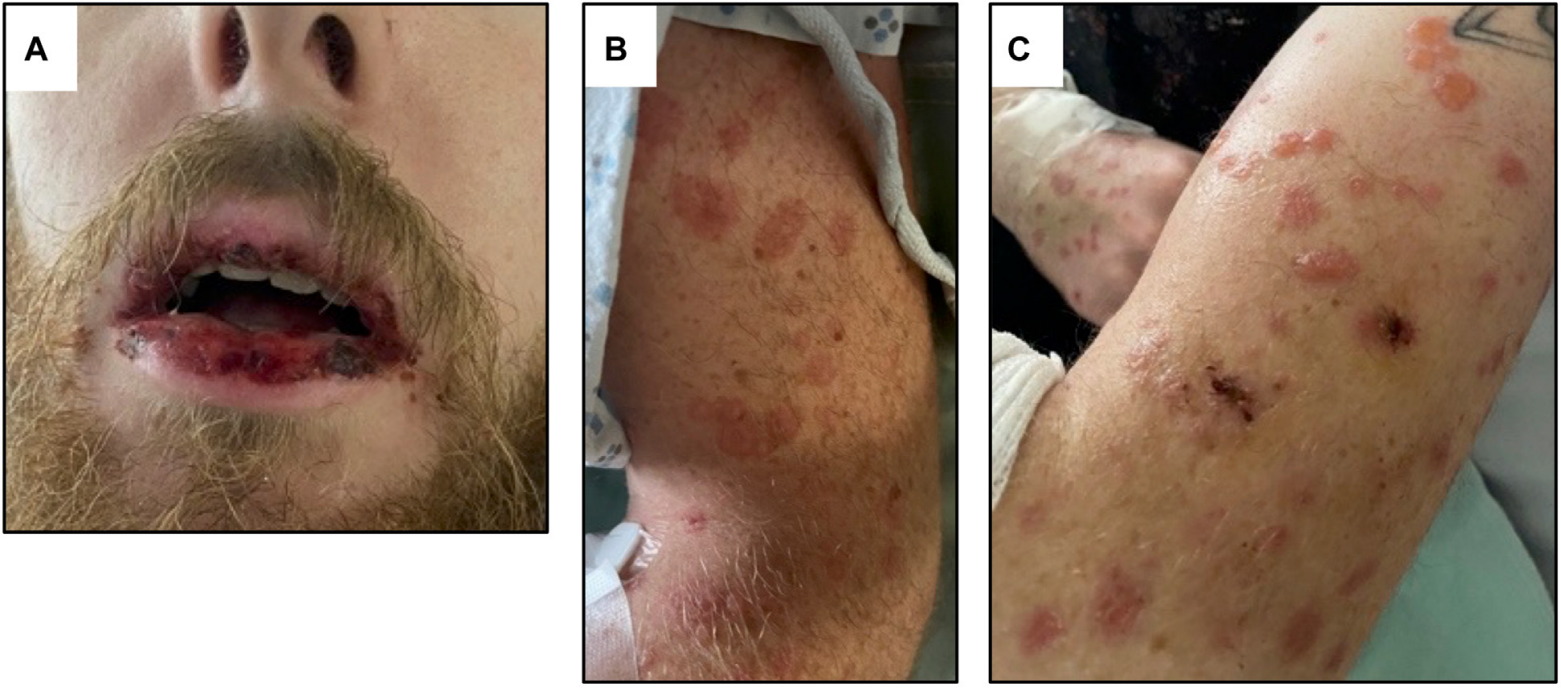
Mucocutaneous findings at initial presentation. **A,** Upper and lower lips with mucosal erosions and hemorrhagic crusting. Pink annular edematous papules, some (**B**) targetoid, (**C**) some bullous, on bilateral arms extending to dorsal hands and bilateral shins extending to ankles with sparing of the face and trunk.

The patient presented to the hospital tachycardic and febrile to 39.6 °C. Exam was notable for conjunctival injection bilaterally, lip mucosal erosions and hemorrhagic crusting (Fig 1, *A*), and pink annular edematous papules, some targetoid, some bullous, confluent in areas, on bilateral arms and shins extending to dorsal hands and ankles with face and trunk sparing (Fig 1, *B* and *C*). On hospitalization day 3 urethral ulceration appeared. Laboratory results revealed elevated C-reactive protein and erythrocyte sedimentation rate, mild leukocytosis, and respiratory virus panel positive for coronavirus (strains 229E, HKU1, NL63, OC43). Chest X-ray was unremarkable. Rheumatologic work-up with antinuclear, double-stranded DNA, extractable nuclear antigen, and antineutrophilic cytoplasmic antibodies, complements 3/4, immunoglobulins, and rheumatoid factor were negative. Ophthalmologic evaluation was inconsistent with uveitis. Infectious work-up showed no evidence of HIV, rubeola, tuberculosis, sexually transmitted or tick-borne diseases, or urine or blood infection. Oral lesion polymerase chain reactions were negative for herpes simplex/varicella, and Epstein-Barr virus titers suggested prior resolved infection. *M pneumoniae* testing showed positive IgG titers (2.15 U/L) but negative IgM titers and oropharyngeal polymerase chain reaction. The patient was monitored off antibiotics with supportive care.

**Fig 2:**
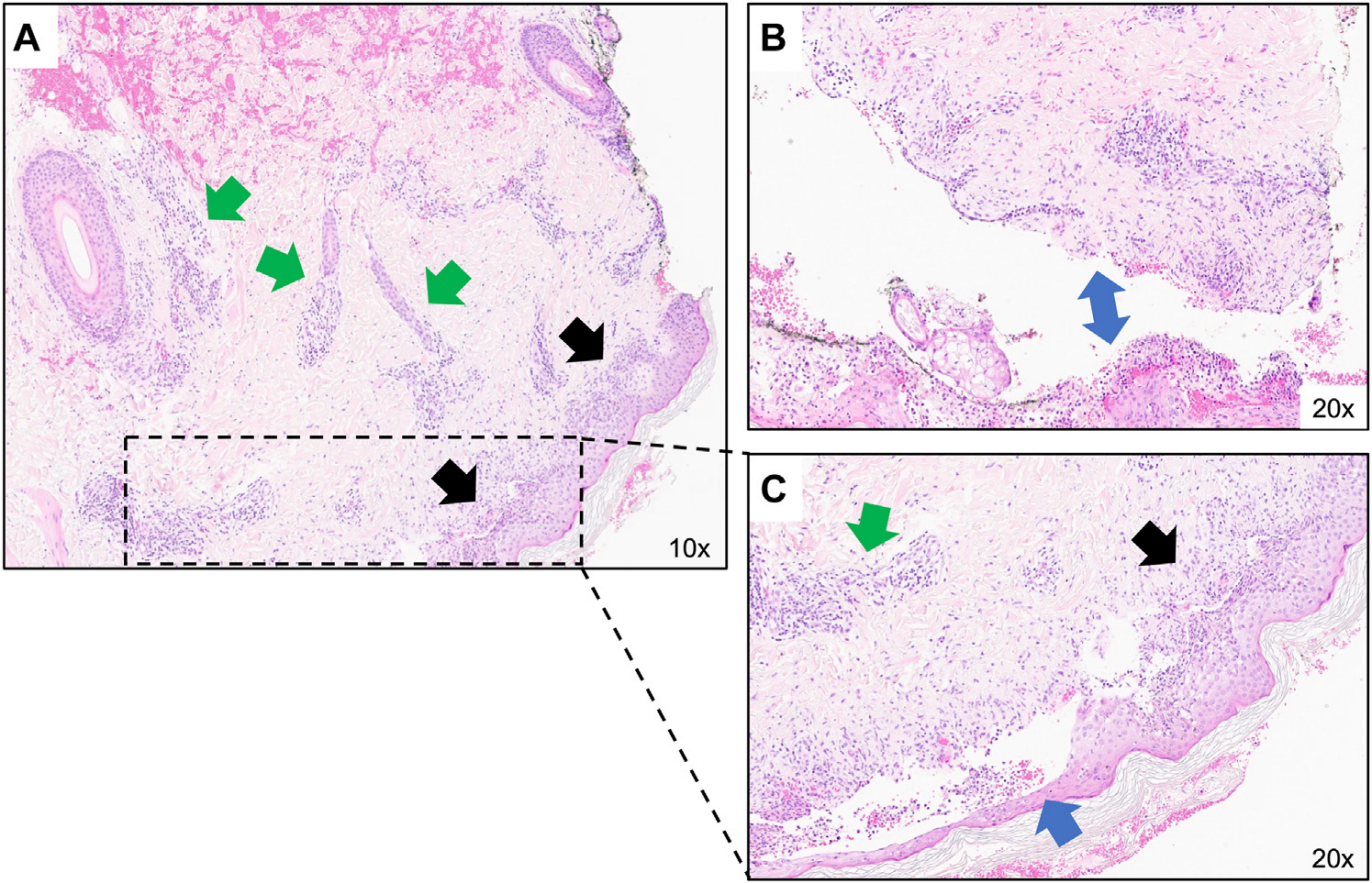
Histochemical evaluation of left arm punch biopsy of targetoid lesion in area of blister formation. There is superficial perivascular and peri-appendageal infiltrate (*green arrows*) as well as lichenoid interface dermatitis (*black arrows*) involving the epidermis base resulting in subepidermal separation (*blue arrows*) (hematoxylin-eosin; (**A**) 10×; (**B**) 20×; (**C**) 20×).

Left arm punch biopsies were obtained, with pathology showing lichenoid interface dermatitis resulting in subepidermal separation consistent with RIME (Fig 2). Direct and indirect immunofluorescence showed no signs of vasculitis, lupus, or autoimmune bullous disease. The patient was treated with daily IVIG for 4 doses and 6 days of IV methylprednisolone 60 mg with clinical improvement, and transitioned to an oral corticosteroid taper on discharge lasting 18 days.

## Discussion

This report describes a young-adult male with a recurrent RIME episode secondary to common coronavirus. He presented with 4 to 5 days of upper respiratory infection symptoms before developing oral, ocular, genitourinary, and cutaneous involvement. Significant overlap exists between RIME and erythema multiforme,^2^ but overall clinicopathologic correlation of multiple mucosal sites involved, greater severity of mucosal involvement than cutaneous, predominance of vesicobullous lesions with rarer targetoid lesions, and palmoplantar sparing was most consistent with recurrent RIME.^2^^,^^9^ Previous descriptions of SARS-CoV-2–associated RIME reported 3 to 14 days of prodromal symptoms averaging 7.71 ± 1.45 days before onset of mucocutaneous symptoms (Table I). These reports describe ocular, cutaneous, or genitourinary involvement in 35.3%, 29.4%, and 58.8% of cases, respectively (Table I). The median age of SARS-CoV-2–associated RIME onset was 23 years, and 88% of cases were male.

**Table I:** Characteristics of patients with coronavirus-associated RIME episode(s)

Authors	Year	PMID	Age (y)	Sex	Time until lesions (d)	Oral, ocular, skin, or genitourinary (GU) involvement	Number of recurrent RIME episodes (episode triggering pathogen)
Mota, et al	2023	36638409	17	Male	7	Oral	0 (COVID-19)
Gimeno, et al^3^	2022	35536675	39	Male	14	Oral, skin, GU	0 (COVID-19)
Song, et al	2021	34515364	16+	Male	3	Oral	4 (*M pneumoniae*, group A strep, influenza A, COVID-19)
van Dam, et al	2023	37258050	Early 30s	Male	7	Oral, GU	4 (*M pneumoniae*, COVID-19, *M pneumoniae*, ?)
Ryder, et al^4^	2021	34692963	17	Male	7	Oral, ocular, GU	0 (COVID-19)
Ortiz and Junkins-Hopkins^5^	2023	36194075	16	Male	5	Oral, skin, GU	0 (COVID-19)
Fan, et al	2022	35875431	16	Male	7	Oral, ocular	0 (COVID-19)
Bowe, et al	2021	34542915	17	Male	8	Oral, GU	0 (COVID-19)
			14	Male	9	Oral, ocular, GU	0 (COVID-19)
Wu, et al^6^	2023	36819988	40	Male	4	Oral, ocular, skin	4 (?, ? ?, COVID-19)
Holcomb, et al^7^	2021	33825803	17	Male	10	Oral, skin, GU	0 (COVID-19)
Bainvoll, et al	2022	–	23	Male	4	Oral	2 (?, COVID-19)
Aw, et al^8^	2023	36340859	25	Female	7	Oral	0 (COVID-19)
			34	Male	8	Oral, ocular, GU	0 (COVID-19)
			25	Female	9	Oral, skin	0 (COVID-19)
			12	Male	8	Oral, GU	0 (COVID-19)
Mahama, et al	2023	36042536	13	Male	14	Oral, ocular, GU	0 (COVID-19)
			Median: 23 y	88% male 12% female	Mean: 7.71 d ± 1.45 (range 3-14)	Oral (100%), ocular (35.3%), skin (29.4%), GU (58.8%)	

*PMID*, PubMed identification; *RIME*, Reactive infectious mucocutaneous eruption.

A novel feature of this case is the inciting infection for the recurrent RIME episode was common coronavirus. RIME is estimated to recur at a rate of 9% to 38%,^10^ and earlier reports of recurrent RIME found the majority developed MIRM initially. This has led to the hypothesis that *Mycoplasma* infection triggers tissue damage: through a direct mechanism involving local release of cytokines, or indirect mechanism in which *Mycoplasma* antibodies provoke immune complex deposition and complement activation.^11^ The present patient had a resolved *Mycoplasma* infection, and the inciting infection of his index RIME episode was SARS-CoV-2. The triggering infection of his second RIME episode was not identified though he reported upper respiratory infection symptoms contemporaneously. In other reports of SARS-CoV-2–associated RIME, we found infection with SARS-CoV-2 caused an index RIME episode in 13 cases and a recurrent episode in 4 cases (Table I).

These reports describe decreasing symptom severity and duration with recurring episodes.^6^ However, our patient experienced worsening recurrent symptoms, suggesting additional factors beyond age and infectious trigger determine severity. Those prone to recurrent RIME may be clinically distinct, and retrospective evaluation could reveal similarities for identifying treatment or screening strategies. Given that MIRM/RIME generally have milder symptoms, supportive care is emphasized and further treatment utilizes immunomodulators including corticosteroids, IVIG, cyclosporine, and tumor necrosis factor inhibitors, although data supporting their use are limited.^2^^,^^8^^,^^12^^,^^13^ A standard of care has not been established for MIRM/RIME or recurrent episodes beyond involving ophthalmology, gynecology, urology, and dermatology services. Resolution of RIME occurs within 1 to 3 weeks of initial mucocutaneous symptoms.^3^, ^4^, ^5^^,^^7^ Our patient received IVIG followed by methylprednisolone and oral taper, with discharge on hospitalization day 9.

In summary, we present the case of a young-adult male with recurrent RIME following infection with common coronavirus. Recurrent RIME remains a novel concept, and our case describes features rarely reported within the literature including: 3-episode RIME recurrence, common coronavirus-triggered recurrence, and increasing severity with recurrence. The patient had no clear previous MIRM episode though had IgGs to *Mycoplasma*, which may represent the predisposing factor responsible for recurrent RIME episodes. We suggest that work-up for patients with a possible presentation of RIME include testing for common and pandemic-associated coronavirus strains among additional evaluation for history of new medications and other respiratory pathogens known to trigger RIME. Future studies identifying commonalities between recurrent RIME cases may shed light on preventive or treatment strategies.

## Conflicts of interest

None disclosed.
